# Effect of Monthly 100,000 IU Vitamin D Supplementation on Falls and Non-Vertebral Fractures

**DOI:** 10.7759/cureus.12445

**Published:** 2021-01-03

**Authors:** Sham Lal Prithiani, Ratan Kumar, Shahid H Mirani, Sadaf Ibrahim, Tanveer Ahmed Ansari, Besham Kumar, Talal Arshad, Syeda M Hassan

**Affiliations:** 1 Internal Medicine, Chandka Medical College, Larkana, PAK; 2 Internal Medicine, Civil Hospital Khairpur, Khairpur, PAK; 3 Surgery, Ghulam Muhammad Mahar Medical College, Sukkur, PAK; 4 Pharmacology, Ziauddin University, Karachi, PAK; 5 Internal Medicine, Pakistan Atomic Energy Commission General Hospital, Mianwali, PAK; 6 Internal Medicine, Jinnah Postgraduate Medical Centre, Karachi, PAK; 7 Internal Medicine, Jinnah Sindh Medical University, Karachi, PAK; 8 Medicine, Jinnah Sindh Medical University, Karachi, PAK

**Keywords:** vitamin d, fractures, falls, elderly

## Abstract

Introduction

Correlation between decreased levels of vitamin D in the blood of elderly patients and incidence of falls and fractures has been assessed in various studies; however, there is still ambiguity in data. In this study, we aim to establish the role of vitamin D supplements in minimizing the burden of falls and non-vertebral fractures in the elderly population in a local setting.

Methods

This single-blind, placebo-controlled randomized interventional study was conducted in the Internal Medicine Department of a tertiary care hospital in Pakistan from March 2018 to July 2020. Patients between the ages of 50 to 75 years were enrolled in the study and were randomly assigned to receive either placebo or 100,000 IU vitamin D oral tablets and were followed over 24 months, with regular follow-ups every three months.

Results

There was no significant difference in the probability of one or more falls for those assigned to the vitamin D group compared to those who received placebo (24.70% vs. 24.85%; hazard ratio [HR]: 0.99; 95% CI: 0.68-1.43). Similarly, the probability of non-vertebral fracture was also non-significant between both groups (4.7% vs. 5.7%; HR: 0.81; 95% CI: 0.32-2.01).

Conclusion

As per the results of this study, vitamin D supplementation had no beneficial effect on the reduction of falls and non-vertebral fractures in elderly patients. Further multi-center studies of longer duration are required to prove the favorable effects of vitamin D supplements.

## Introduction

Vitamin D plays a crucial role in maintaining the mineralization of the skeletal bones in the human body. Exposure to sunlight leads to the production of vitamin D in the skin, which is metabolized to its active form, 1,25-dihydroxyvitamin D, by the liver and kidney. Vitamin D deficiency is being recognized as a major cause of metabolic bone diseases in the elderly, leading to falls and fractures [1].

The role of vitamin D supplementation has already been recognized in the treatment and prevention of rickets in children and osteomalacia in adults [2,3]. The dose that is recommended for daily use is 400 international units (IU) of vitamin D since birth [2,3]. Chapuy et al. found that when vitamin D was administered to elderly women for 18 months, it resulted in a 43% decrease in hip fractures and a 32% decrease in other non-vertebral fractures [4].

As age increase, multiple factors such as sun exposure, dietary modifications, physical inactivity, and pathophysiological alterations lead to decrease intestinal and renal calcium absorption, decreased 25-hydroxyvitamin D (25-OHD) hydroxylation by kidneys, and decreased levels of parathyroid hormone (PTH), which affect the bone mineral density [5].

Correlation between decreased levels of vitamin D in the blood of elderly patients and incidence of falls and fractures has been identified, but various clinical trials and meta-analyses have not been able to establish a clear role of vitamin D supplements in reducing the risk [6-9]. In this study, we aim to establish the role of vitamin D supplements in minimizing the burden of falls and non-vertebral fractures in the elderly population.

## Materials and methods

This single-blind, two-arm, placebo-controlled interventional study was conducted in the Internal Medicine Department of a tertiary care hospital in Pakistan between March 2018 and July 2020. Patients were enrolled until June 2018, and follow-up was completed by July 2020. A total of 400 participants of either gender between 50 and 75 years of age were enrolled in the study after obtaining informed consent. Participants were enrolled through consecutive convenient non-probability sampling.

Participants already on vitamin D supplement and those with balance disorder, psychiatric disorder, a history of hypercalcemia, nephrolithiasis, and parathyroid disease were excluded from the study. Participants were randomly allocated using online software Research Randomizer (https://www.randomizer.org/) to either the vitamin D group (100,000 IU oral monthly) or the placebo group. Both vitamin D tablets and look-alike placebo were provided at the clinic in the same opaque bottle for the next three months at every follow-up.

Age, gender, smoking status, body mass index (BMI), and frequency of physical activity were noted using a self-structured questionnaire. Physical activity was defined as one to two days of exercise in one week. Blood was taken through phlebotomy and sent to a laboratory to determine vitamin D levels. Patients with normal vitamin D levels were excluded from the study.

Participants were followed for 24 months, with visit to the outpatient department after every three months, during which they were given either vitamin D or placebo based on their group for the next three months. Participants were phoned every month to remind them about taking their medicine and were also inquired about any fall or fracture in the previous month. A total of 24 and 21 participants were lost to follow-up from the intervention and placebo groups, respectively. Six patients died in each group. Data of participants who completed the study were included in the final analysis. The primary outcome for this study was the number of falls and non-vertebral fractures. Non-vertebral fracture was defined as fracture occurring in any part of body other than the spine and skull.

Statistical analysis was conducted using SPSS Version 23.0 (IBM Corp., Armonk, NY, USA). Continuous variables were analyzed using descriptive statistics and were presented as means and standard deviations (SDs), whereas categorical variables were analyzed using t-test and chi-square tests, as appropriate. Hazard ratio (HR) was used to compare the interventional group with the placebo group. A p-value of less than 0.05 meant that there was significant difference between two groups and null hypothesis was void.

## Results

Characteristics between the intervention group and the placebo group were comparable (Table 1).

**Table 1:** Characteristics of the Interventional and Placebo Groups SD, standard deviation; BMI, body mass index; NS, non-significant

Characteristics	Intervention Group (n = 170)	Placebo Group (n = 173)	p-Value
Age in years (mean ± SD)	62 ± 10	61 ± 11	NS
Male (%)	48.2	46.2	NS
BMI greater than 25 kg/m^2^ (%)	52.3	54.3	NS
Current smokers (%)	30.0	34.6	NS
Physically active (%)	15.2	16.1	NS
Vitamin D, ng/mL (mean ± SD)	22.45 ± 5.81	23.22 ± 5.62	NS

The probability of reporting one or more falls was similar for those assigned to the vitamin D group compared to those assigned to the placebo group (24.70% vs. 24.85%; HR 0.99; 95% CI: 0.68-1.43). Similarly, the probability of non-vertebral fracture was also similar between both groups (4.7% vs. 5.7%; HR: 0.81; 95% CI: 0.32-2.01) (Table 2).

**Table 2:** Outcome of the Interventional and Placebo Groups

Outcome	Intervention Group (n = 170)	Placebo Group (n = 173)	Hazard Ratio (95% CI)	p-Value
All falls	42 (24.70%)	43 (24.85%)	0.99 (0.68-1.43)	0.97
Non-vertebral fracture	8 (4.7%)	10 (5.7%)	0.81 (0.32-2.01)	0.65

## Discussion

Vitamin D is commonly prescribed for the treatment and prevention of rickets and osteomalacia in children and adults, respectively [2,3]. Vitamin D plays a major part in the development, growth, and mineralization of the skeletal system of all ages. Its deficiency leads to reduced intestinal calcium absorption that sends a signal to the parathyroid gland to secrete PTH. PTH increases calcium levels by stimulating osteoclasts, resulting in resorption of bones, which exacerbates osteoporosis in older adults [1].

Various studies have been conducted to study the correlation between vitamin D levels and incidence of falls and fractures. Fosnight et al. reviewed this correlation and found a significantly positive relationship between vitamin D supplementation with either cholecalciferol (vitamin D3) 700 IU/day or greater or with ergocalciferol (vitamin D2) 800 IU/day or greater and decreased risk of falls [10]. In their meta-analysis, Bischoff-Ferrari et al. concluded that 700-800 IU/day of vitamin D reduced the relative risk of hip fractures by 26% and any non-vertebral fractures by 23% in elderly people, with no significant benefit observed in participants receiving 400 IU/day of vitamin D [11]. Bolus doses of vitamin D given annually (at a dose of 300,000 IU or 500,000 IU) or monthly (at a dose of 24,000 IU or 60,000 IU) have shown to significantly increase incidence of falls and fractures associated with >40-45 ng/mL (100-112.5 nmol/L) levels of 25-OHD in elderly individuals [12].

In contrast, in this study, the incidence of one or more falls and non-vertebral fracture was similar for those assigned to the vitamin D group compared to those assigned to the placebo group (24.70% vs. 24.85% and 4.7% vs. 5.7%). Similar results were reported by a randomized, double-blind, placebo-controlled vitamin D assessment (ViDA) trial, which was conducted on 5,110 participants with a mean age of 65.9 years. In that study, 100,000 IU monthly dose of vitamin D did not prevent falls or non-vertebral fractures [6]. The American Geriatrics Society recommended 4,000 IU daily for fall prevention, especially in those with a history of falls [13]. However, Smith et al. in their randomized clinical trial suggested that incidences of fall might be related to vitamin D dose. They concluded that there was a significant decrease in the incidence of fall when participants took a medium (1,600, 2,400, 3,200 IU) daily dose of vitamin D (p = 0.020) and no decrease in falls in those taking low (400, 800 IU) or high (4,000, 4,800 IU) doses of vitamin D. Fall rates on high doses increased more when compared to medium doses as the serum 25-OHD levels exceeds 40-45 ng/mL [14]. In another randomized controlled trial, administration of oral 150,000 IU vitamin D3 for three months had neither a beneficial nor any adverse effect on falls or physical function in 686 women over the age of 70 years [15].

To the best of our knowledge, this is the first study that assesses the role of high-dose monthly vitamin D in reducing fall and non-vertebral fracture. The study has its limitation as well. The study was conducted at a single center, which may have reduced the diversity of sample. Further large-scale multi-center studies are needed to understand the role of different doses of vitamin D in reducing falls and fractures.

## Conclusions

Vitamin D has a well-established role in bone mineralization and strength and has a significant effect on calcium and phosphate metabolism and muscle function due to which its beneficial effect on risk of falls and fractures is expected. However, the data from our clinical study indicate that the supplementation of vitamin D over a period of two years had no significant role in the reduction of falls and non-vertebral fractures. However, further large-scale studies are required to study the association between incidence of falls and different doses of vitamin D.
